# Editorial: Wound management and healing in space

**DOI:** 10.3389/fbioe.2022.1078986

**Published:** 2022-11-23

**Authors:** Monica Monici, Jack J. W. A. van Loon, Alexander Choukér, Carlo S. Iorio

**Affiliations:** ^1^ ASAcampus Joint Laboratory, ASA Res Div., Department of Experimental and Clinical Biomedical Sciences “Mario Serio”, University of Florence, Florence, Italy; ^2^ Dutch Experiment Support Center (DESC), Amsterdam Bone Center (ABC), Amsterdam University Medical Center, Location VU University Medical Center (VUmc) and Academic Centre for Dentistry Amsterdam (ACTA), Department of Oral and Maxillofacial Surgery/Oral Pathology, Amsterdam, Netherlands; ^3^ European Space Agency (ESA), European Space Research and Technology Centre (ESTEC), TEC-MMG, Noordwijk, Netherlands; ^4^ Laboratory of Translational Research “Stress and Immunity”, LMU University Hospital Munich, Munich, Germany; ^5^ Centre for Research and Engineering in Space Technologies - CREST, Service de Aero-thermo-mechanique - ATM, Université libre de Bruxelles, Bruxelles, Belgium

**Keywords:** wound healing, tissue repair and regeneration, space, space exploration, space exposome

Life without wound healing (WH) is impossible. To repair injured tissues is a biological process of crucial importance because it restores the tissue´s homeostasis and hereby the integrity of the body. WH is an extremely complex process. It consists of a succession of events that have as protagonists different cell populations whose behavior is strictly regulated qualitatively, quantitatively and temporally by multiple biochemical and biophysical factors.

Despite the large amount of studies carried out and the remarkable progress recently made in understanding the mechanisms underlying WH, there are still many knowledge gaps to be filled in order to progress in the development of effective therapeutic strategies, which aim to promote tissue regeneration instead of scarring.

This Special Issue on “Wound Management and Healing in Space” aims to address all the factors that could affect WH in this exposome of the hostile environment, as characterized by microgravity, radiation, conditions of isolation and confinement. In Space the human body undergoes an adaptation process that is characterized by pathophysiological alterations that could modify the resilience of the organism and its ability to respond to injuries. Furthermore, according to our present knowledge, the behavior of cell populations involved in WH is altered in Space. Very likely, these changes will have an impact on WH in space exploration.

In future space exploration missions beyond low Earth orbit (LEO), already small wounds can affect the mission performance and can last longer than expected. The management of serious wounds however, both traumatic and surgical, ulcers, and burns could be particularly challenging and mission ciritical due to the impossibility of carrying out a rapid medical evacuation to Earth. Also the delay in communications limits the remote assisted procedures and compromises the ability to guide the crew efficiently. Therefore, Space Agencies have included WH in the context of spaceflight among the potentially critical problems to be approached for planning future deep space missions.

A perspective paper (Puhl et al.) included in this collection of manuscripts reports the approach of the European Space Agency (ESA), that started with the setup of a specific Topical Team (TT) of experts to identify potential concerns about WH in Space, draw up recommendations and suggest countermeasures. Currently, ESA is supporting research projects focused on specific aspects of WH and tissue regeneration in Space, and, in future perspective, it is building a 3D bioprinting system and a bioreactor for the maturation of tissue-constructs on the ISS. These facilities are a crucial starting point to enable advanced strategies for tissue regeneration and development of personalized grafts for crew members if serious injuries were to happen during long-term deep space exploration missions.

An important topic connected to WH and hemorrhage is hemostasis and platelet function, which also contributes to other biological processes through the release of growth factors and many other molecules. The paper by Locatelli et al. reviewed the very few studies on platelet function in Space. The results of these studies demonstrated that microgravity affects platelet’s number and function, thus increasing the risk of hemorrhages and contributing to delay WH. However, Platelet Rich Plasma (PRP), although in simulated microgravity it proved less effective than in normogravity, could be evaluated as a countermeasure to prevent WH delay.

After hemostasis, the process of WH proper begins, classically divided into the inflammatory, proliferative, and remodelling phases. Fibroblasts have a significant role in all the three phases and they are the main protagonists of the remodelling phase. Moreover, they orchestrate the whole healing process through cross talk with immune cells, endothelial cells, and keratinocytes. Due to this central role in the process, the studies on the effects induced by real and modeled microgravity on fibroblast functions involved in WH have been reviewed by Cialdai et al. to define the gaps of knowledge about fibroblast function in Space and also to provide cues for developing adequate countermeasures. Interestingly, some microgravity-induced alterations of fibroblast function are similar to fibroblast dysfunctions observed in impaired WH on Earth.

Also research on the behavior of endothelial cells in weightlessness has been reviewed, as endothelial cells are responsible for neoangiogenesis during the proliferative phase of WH (Morbidelli et al). Angiogenesis output comes from a finely regulated balance between pro- and antiangiogenic factors, in order to avoid insufficient or excessive nonreparative neovascularization. The understanding of the factors and mechanisms that control angiogenesis and their changes in unloading conditions can help to design countermeasures to optimize neoangiogenesis in case of traumatic injury or surgical wounds during missions.

A mini-review (Bacci and Bani) on the effects of unloading conditions on epidermis and keratinocytes, that are responsible for re-epithelialization, shows that epidermal stem cells cultured in simulated microgravity undergo enhanced proliferation and viability and reduced terminal differentiation than under normal gravity. In the meantime, microgravity also triggers epithelial-mesenchymal transition of keratinocytes, promoting a migratory behavior. However the cross-talk between fibroblasts and keratinocytes is impaired and epidermal repair is delayed. These results confirm that WH is an “ensemble” that needs a strict regulation as regards timing and players, respectively.

One of the most important mechanisms in WH regulation is apoptosis, or programmed cell death. Apoptosis enables orchestrated cell removal in wounded or infected tissues. A dedicated review (Riwaldt et al.) provides an overview of alterations in the behavior of cutaneous cell lineages under microgravity, in regard to the impact of apoptosis in WH. Moreover, the current knowledge about WH in Space and simulated microgravity with respect to apoptosis and available therapeutic strategies is discussed, and the opportunity to use microgravity to obtain new insights into the role of apoptosis in WH is considered.

Correct WH evolution is also affected by systemic conditions. The association of insulin resistance and WH impairment may be hypothesized from some dysmetabolic conditions, like the metabolic syndrome, type 2 diabetes mellitus and abdominal/visceral obesity, where derangement of glucose and lipid metabolism, greater low-grade inflammation, altered adipokine secretion and adipocyte dysfunction converge to produce systemic effects that also negatively involve WH. Interestingly, chronic low-grade inflammation and insulin resistance appear to be pivotal events linking many of the pathophysiological alterations induced by spaceflight. Based on these considerations, one of the papers of this collection is devoted to discuss the pathophysiological links between microgravity-associated insulin resistance and impaired WH (Strollo et al.).

The microbial populations settled on skin, space modules, and in space suits can also play a significant role in WH. A paper of this special issue (Marvasi et al.) discusses a perspective that includes four domines for applying skin microbiota to WH in Space: 1) the natural antimicrobial properties of the skin microbiota, 2) the cross-talk between skin microbiota and immune system during WH, 3) the contribution of the microbiota in precision medicine, and 4) the role of gut-skin and gut-brain axes. A stronger understanding of the connections among bacteria, fungi, host immune system, and host metabolism could help improving WH in Space and on Earth.

Two main objectives of the studies on WH are: 1) find strategies leading to tissue regeneration; 2) create tissue substitutes to replace damaged tissues and support their functions. The achievement of these objectives is particularly important to manage WH in Space. Three-dimensional (3D) bioprinting (BP) might offer a solution, providing 3D tissue constructs, which can serve as models in basic research as well as in the development of transplantable skin grafts. The perspective paper dealing with this topic provides an overview of the state of the art of skin BP and approaches to establish this additive manufacturing technology in Space. In addition, the several advantages of BP for utilization in future manned space missions are highlighted (Cubo-Mateo and Gelinsky).

A new autologous micrografting (AMG) technology is described in a manuscript (Aliberti et al.) that shows how its use can remove the limitations associated with autograft transplants (e.g., risk of infections, secondary diseases, and low compliance for the patient). Among several re-epithelialization technologies developed to establish a physiological WH process, it has been demonstrated that the AMG technology is able to respond to the principal limitations of the current gold standard approach to autologous grafting. This includes the need to use large quantities of tissues, long sample preparation time, and long-term hospitalization. The proposed AMG technology plays a key role in the reepithelialization stage by modulating the genes responsible for angiogenesis and cell migration, including the migration of fibroblasts.

Temporary storage of nasal tissues and nasal cell sheets is an important issue in regenerative medicine. One of the presented papers (Kasai et al.) reports a study that investigated the preservation of chilled and frozen nasal tissues and expiry dates of ready-to-use nasal cell sheets. The results show that nasal tissues can be stored temporarily in refrigerators or deep freezers, and Hank’s balanced salt solution (HBSS) can be used for preservation of ready-to-use cell sheets for a few days. *In vitro* cell sheet grafting assays demonstrated that cell sheets stored in HBSS for 2 days adhered to collagen gel and expanded normally.

An interesting original research by Leyi Xue et al., which is included in this paper collection, focuses on the development of Artemisia argyi plant extract (AE) loaded composite hydrogel scaffold based on methacrylate gelatin (GelMA)/methacrylate hyaluronic acid (HAMA) and mesoporous silica nanoparticle (MSN) as sustained-release drug carrier vehicles for the treatment of chronic wounds. In *vitro* and *in vivo* (animal model) experiments the AE loaded hydrogel showed stable rheological properties, suitable mechanical properties, appropriate biodegradability and biocompatibility, swelling, sustained capacity to release AE, which confers significant antibacterial and anti-inflammatory effects. Moreover, the AE-loaded hydrogel was able to induce in macrophages the transition from M1 to M2 phenotype and promote WH modulating the expression of pro- and anti-inflammatory cytokines.

Distinct physical factors existing in the cellular microenvironment are crucial to the biological homeostasis of stem cells. While substrate stiffness and orientation are known to regulate the mechanical remodelling and fate decision of mesenchymal stem cells (MSCs) separately, it remains unclear how the two factors are combined to manipulate their mechanical stability under gravity vector. An original paper studied these combined effects and showed that: i) in the different conditions, MSC mechanical stability is reached through changes in the cytoskeletal networks of actin and vimentin; ii) cell morphology and focal adhesion are mainly affected by substrate stiffness; iii) mechanistically, in the different stiffness conditions, the cell tends to be stabilized *via* β1 integrin–focal adhesion complexes–actin mechanosensitive axis (Zhang et al.).

In remote environments, such as deep space, where diagnostic tools and medical surveillance are scarce, the monitoring of clinical parameters could become crucial to timely detect life-threatening conditions. Nowadays, some signals can be measured using wearable technology. However, often the low quality of the recordings leads to wrong conclusions. Therefore, it is important to determine which parts of the signal are of sufficient quality. The results of a study by Rozo et al. on the quality assessment of respiratory signals obtained from wearable sensors show that with pre-trained machine learning classifiers in conjunction with data augmentation and transfer learning, it is possible to properly identify clean and noisy respiratory bioimpedance signals. These findings could be beneficial for the steps of data processing and connection with decision support systems when designing new bio-monitoring devices for space exploration.

Pantalone et al. described accordingly the possibility, even if remote, that serious traumatic events occur or there is a need for surgical treatments aboard a spacecraft. In this case, hemorrhage can be a life-threatening condition. Although the consequences of a haemorrhage during space flight are quite difficult to predict, the different aspects of hemorrage in Space and possible countermeasures are reviewed.

Wound management requires the development of reliable wound monitoring systems to facilitate the assessment and proper care of wounds in isolated environments, such as Space. An original study by Miskovic et al., which aims to develop a device for real-time *in-situ* wound temperature monitoring, provides a full characterization of a sensing element composed of thermotropic liquid crystals arrays embedded between two elastomer layers, and discusses how such a system compares against infrared thermography (non-local measurements), a technique commonly used to measure temperature distribution at the wound site.

The collection of papers in this special issue derives largely from the activities carried out within the ESA-TT “Tissue Healing in Space: Techniques for Promoting and Monitoring Tissue Repair and Regeneration” and the ESA supported-Microgravity Application Program (MAP) WHISPER Project “Wound Healing in Space: Problems and Perspectives for Tissue Regeneration and Engineering”.

The guest editors thank all the authors who contributed to the Special Issue on “Wound Management and Healing in Space” with their very interesting manuscripts and we hope the readers will share this view and future studies will contribute to a better understanding of wound behavior, and hence wound treatment, in human space exploration missions.

